# Advancing precision medicine through the integration of clinical cardiovascular genetics - An Asian perspective

**DOI:** 10.1016/j.gimo.2024.101877

**Published:** 2024-07-24

**Authors:** Iswaree D. Balakrishnan, Yasmin Bylstra, Nikki Fong, Nellie B.S. Chai, Sylvia Kam, Chun Yuan Khoo, Laura L.H. Chan, Angela S. Koh, Hak Chiaw Tang, Eric Lim, Ju Le Tan, Weng Khong Lim, Chee Jian Pua, David Sim, Stuart A. Cook, Ee Shien Tan, Khung Keong Yeo, Saumya S. Jamuar

**Affiliations:** 1 Department of Cardiology, National Heart Centre Singapore, Singapore; 2 SingHealth Duke-NUS Genomic Medicine Centre, Singapore; 3 SingHealth Duke-NUS Institute of Precision Medicine, Singapore; 4 Genetics Service, Department of Paediatrics, KK Women’s and Children’s Hospital, Singapore; 5 Cardiovascular and Metabolic Disorders Program, Duke-NUS Medical School, Singapore; 6 Cancer & Stem Cell Biology Program, Duke-NUS Medical School, Singapore; 7 Laboratory of Genome Variation Analytics, Genome Institute of Singapore, Agency for Science, Technology and Research, Singapore; 8 National Heart Research Institute Singapore, National Heart Centre Singapore, Singapore

**Keywords:** Asian population, Cardiovascular genetics, Genetic counselling, Precision medicine

## Abstract

**Purpose:**

The integration of cardiovascular genetic (CVG) testing into clinical practice is gaining recognition, but its implementation in the Asian setting has not been widely reported. We present our experience developing a clinical CVG service and analyze its impact on patient care at our center.

**Methods:**

In 2020, the National Heart Centre Singapore collaborated with SingHealth Duke-NUS Genomic Medicine Centre, to establish a comprehensive clinical CVG service. We retrospectively gathered details regarding referral indication and the clinical utility of genetic counseling and testing.

**Results:**

Over a period of 2.5 years, 113 patients aged 17 to 94 years, were seen by the CVG team. The cohort included 79 males and 34 females: 82 of Chinese ancestry, 11 Indian, 7 Malay, and 13 from other ancestries. The most common reason for referral was for cardiomyopathy, followed by aortopathy. After clinical evaluation, 98 patients were offered genetic testing, of which 63 (64%) patients proceeded with genetic testing (diagnostic testing *n* = 51, predictive testing *n* = 10, familial segregation analysis *n* = 2). Eleven patients were referred for continuation of care. Overall, CVG testing added value to the care of 44 patients by clarifying clinical diagnosis, ruling out inherited cardiac disorders, aiding variant of uncertain significance resolution, and/or facilitating cascade testing.

**Conclusion:**

Our pilot initiative has provided insights into the practical value, obstacles, and opportunities for developing a clinical CVG service. The establishment of our clinical CVG service not only enhanced patient care but also demonstrated its scalability through collaborative partnerships with domain experts.

## Introduction

Cardiovascular genetic (CVG) testing, as a subset of precision medicine, has received endorsement and is recommended as the standard of care in recent cardiology clinical practice guidelines.^1^, ^2^, ^3^, ^4^, ^5^ Clinical CVG testing serves 3 primary functions: first, it clarifies clinical or phenotypic diagnoses; second, it informs clinical decision-making; and third, it enables the identification of asymptomatic at-risk relatives through cascade testing.^5^

Despite its established benefits, the implementation of clinical CVG genetic testing has been marked by inertia in the Asian setting.^6^^,^^7^ This can be attributed in part to a lack of awareness and education among health care professionals^8^^,^^9^ and the general public,^10^ deficiencies in infrastructure and expertise^11^^,^^12^ required to operate a clinical service, and, cost and regulatory challenges.^13^^,^^14^

In 2018, the Ministry of Health (MOH), Singapore promulgated guidelines on the provision of clinical genomic and genetic services laying down the requirements, such as mandatory pre and post-test genetic counseling for all patients undergoing genetic testing using next-generation sequencing.

SingHealth Duke-NUS Academic Medical Centre (AMC) is a pioneering collaboration between Singapore’s largest health care group and one of our medical schools.^15^ The AMC was established to deliver high-quality and accessible medical care. The AMC operates within a robust framework that includes 15 dynamic academic clinical programs. These programs, in partnership with leading tertiary institutes, such as National Heart Centre Singapore (NHCS), National Cancer Centre Singapore, and KK Women’s and Children’s Hospital, harness the expertise of the various specialties to advance health care delivery through research, education, and innovation.

In 2019, in view of the increasing use of genomics in clinical care as well as the MOH guidelines, the AMC leadership endorsed the establishment of SingHealth Duke-NUS Genomic Medicine Centre (SDGMC) to develop clinical genomic services at all the tertiary institutes, including our cardiology center, NHCS.

As precision medicine gained ground on a national scale in Singapore, NHCS embraced the opportunity to enhance the integration of clinical CVG into our range of services. In collaboration with SDGMC and with provisional approval from MOH, we established our CVG service in 2020. This initiative aimed to increase CVG literacy among physicians, consolidate previously disparate processes, develop cardiologists’ expertise in genomic medicine, and optimize organizational frameworks to ensure effective, coordinated, and value-driven integration of CVG into clinical practice. This required robust collaboration among stakeholders, including cardiologists, geneticists, other medical specialists, genetic counselors, and regulatory bodies.

In this paper, we share how we developed a comprehensive clinical CVG service, and highlight the significance of genetic counseling and testing and its influence on the delivery of clinical care and patient outcomes at our center.

## Materials and Methods

This study is a retrospective observational review of medical records of patients who were seen by the CVG service from December 2020 to June 2023. We retrospectively collected patient demographics, the clinical utility of genetic counseling, testing, and clinical outcomes. We used descriptive statistics for our analysis. All patient details were redacted to ensure confidentiality. This study has been reviewed by the Centralised Institutional Review Board, and it was determined that the application does not require further ethical deliberation, because this application involves de-identified data.

### Developing the infrastructure for our CVG service

National Heart Centre Singapore is a national and regional referral center for cardiovascular diseases. We offer a comprehensive range of cardiac services, including preventive, diagnostic, therapeutic, and rehabilitative care. As the only heart and lung transplantation center in Singapore, we specialize in advanced treatments for complex cardiovascular conditions. Each year, we handle over 120,000 outpatient consultations, 9000 interventional and surgical procedures, and 10,000 inpatients. The medical team comprises over 140 professionals, including cardiologists, cardiac surgeons, radiologists, and anesthesiologists.

Before the introduction of our pilot CVG service, there was no dedicated clinical CVG service. However, there were certain elements such as a cardiogenomics research laboratory, albeit with a primary research focus. On the clinical front, the provision of genetic service was disjointed with specialists working within their own domains of expertise. For example, electrophysiologists and heart failure cardiologists were utilizing genetic testing independently, without a structured framework, insufficient pre and post-test genetic counseling, and no data collection measures were in place.

In collaboration with SDGMC, a hub and spoke model was established in 2020, bringing together where clinical geneticists (n = 7) and genetic counselors (n = 9) from other specialty centers to partner with NHCS to provide clinical genomic services. This partnership formed the basis of the comprehensive and complete clinical genomic service leveraging each institution’s strength. The scope of the clinical CVG service was broadly classified into 5 domains (Table 1).

**Table 1:** Types of cardiovascular conditions seen at the CVG service

Cardiovascular Condition	Example
Cardiomyopathy	Dilated, arrhythmogenic, hypertrophic cardiomyopathies
Aortopathy	Marfan, Ehlers-Danlos, Loeys-Dietz syndromes
Arrhythmogenic disorders	Long QT, Brugada syndromes
Lipid disorders	Familial hypercholesteremia
Other cardiovascular conditions	Pulmonary arterial hypertension, complex congenital heart disease

Through collaborative efforts, we developed an operational framework for a clinical service by combining expertise in cardiovascular disease, genomic technologies, and genetic counseling. Initially led by a clinical geneticist and genetic counselor, we subsequently enhanced the service with the inclusion of a cardiologist specializing in inherited cardiac conditions, thereby increasing the relevance of genetics in the context of cardiovascular diseases. The multidisciplinary nature of the service facilitated greater visibility of the clinical and operational challenges. This, in turn, prompted meaningful communication between the different teams leading to iterative improvements in various processes.

Simultaneously, we worked on increasing the awareness and clinical utility of CVG testing among physicians and patients through educational seminars and public forums. Although Singapore does not have a formal training program in clinical genetics, clinical geneticists who trained in overseas centers including Australia (n = 3), the United Kingdom (n = 1), and the United States (n = 3), worked with SDGMC to develop courses^16^ in clinical genomics to upskill healthcare professionals within the local ecosystem, including NHCS. We also updated existing patient education resources with a focus on the relevance of genomics in inherited cardiovascular diseases.

Genetic testing was conducted through established overseas clinical genetic testing laboratories and a local clinical genetic testing laboratory with which we had signed service-level agreements. Looking ahead, we are planning to transform our cardiogenomics research laboratory into a clinical-grade facility to enable in-house genetic testing and advance our clinical services.

### Clinical care model

The CVG service is committed to offering a seamless and personalized experience for every patient, starting with a thorough pre-clinic preparation conducted by the core CVG team comprising cardiologists specializing in inherited cardiac conditions, clinical geneticists, and genetic counselors. The genetic counselor does the intake and summarizes the notes relevant to the genetic consultation. Before each clinic session, the team familiarizes themselves with the patient’s history and makes preliminary decisions on the choice of genetic testing, thereby allowing sufficient time for a detailed family history gathering and genetic counseling during the clinic session. A key focus of the pre-clinic session is carefully curating genetic variants from available reports to determine variant classification and establish genotype-phenotype correlation. This takes into account local variant prevalence data,^17^ which may not be captured in other population databases such as gnomAD. Where further review is needed for variant classification, expertise from clinician-scientists and bioinformaticians is sought.

During the CVG clinic session, which is jointly conducted by the core CVG team, patients undergo a comprehensive assessment. This involves a review by the cardiologist to establish or confirm the phenotype. In accordance with ministry guidelines, all patients receive pre-test genetic counseling to ensure they are fully informed about the genetic testing process and its potential implications. Post-test genetic counseling is also provided to patients who undergo genetic testing, helping them interpret test results and plan appropriate next steps. Where genetic testing yields positive results, the CVG team takes proactive steps to facilitate predictive testing for at-risk family members.

All data collected during this process were securely stored in a database for audit purposes, maintaining strict confidentiality. Additionally, regulatory clearance to establish a CVG registry, which will contribute valuable information to enhance and better understand the local genomic diversity, is underway.

### Genetic testing approach

The choice of genetic testing was guided by patients’ phenotype for diagnostic testing, whereas variant-specific testing was used for predictive testing. Almost all patients, except 2, underwent genetic testing with Invitae Corporation’s cardiovascular disorder panels or connective tissue disorders (CTD) panel. The latter was utilized for patients presenting with the predominant musculoskeletal manifestation of CTD, with or without aortopathy. One patient underwent exome sequencing and mitochondrial genome testing through PreventionGenetics, whereas another patient had single transthyretin (*TTR*) gene testing conducted at a local clinical genetic testing laboratory.

## Results

**Figure 1:**
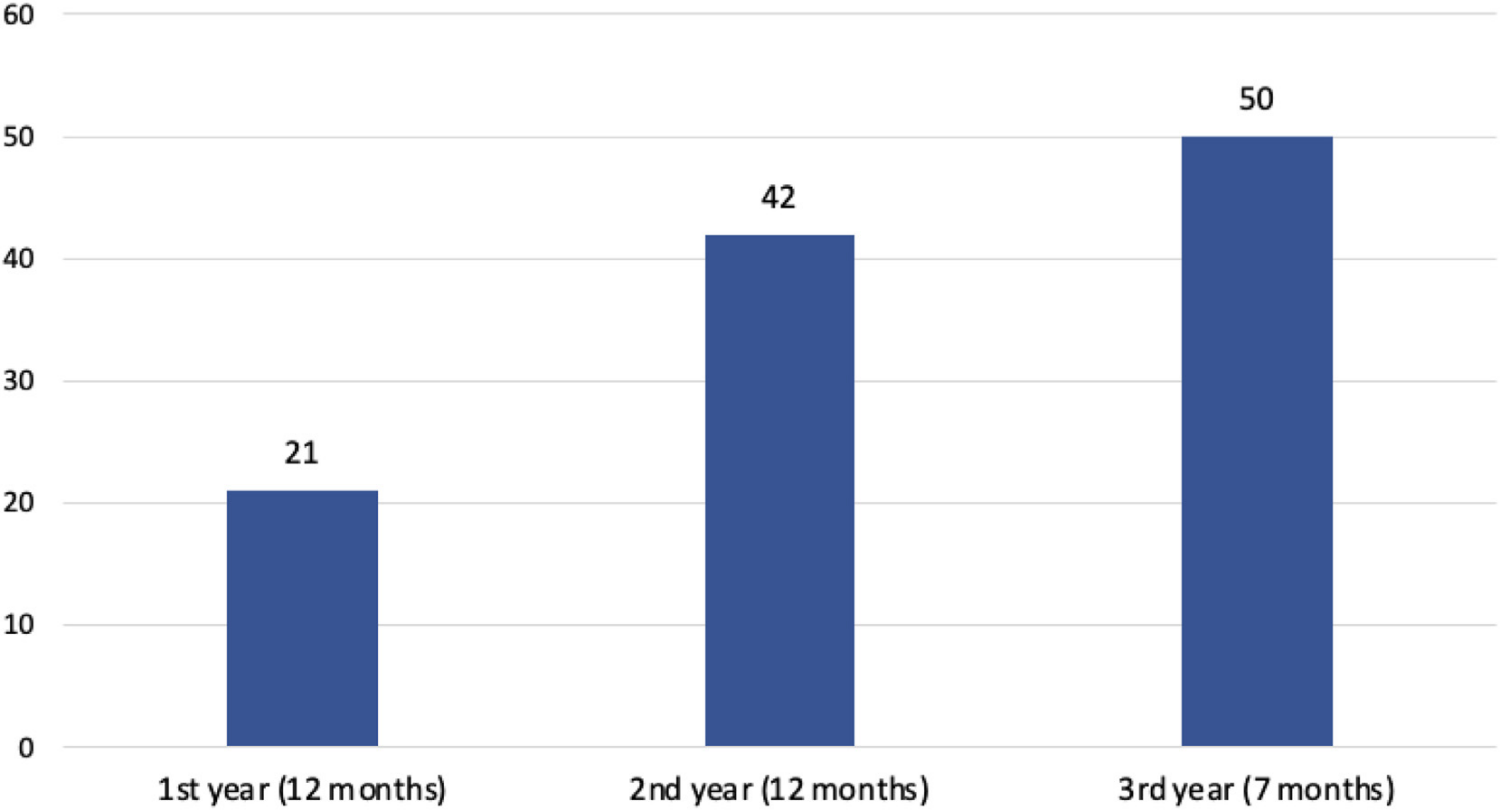
**Number of referrals to the cardiovascular genetics service**.

Over a period of 31 months (December 2020 to June 2023), a total of 113 patients were seen by the CVG team. The majority of patients (95%) were reviewed at the CVG outpatient clinic, and the remaining were seen as inpatient referrals. Patient demographics and referral characteristics are detailed in Table 2. Since the advent of the CVG service, the number of referrals has steadily increased over the years (Figure 1).

**Table 2:** Patient demographics and referral characteristics

Number of patients (*n*)	113
Age – years, range, average	17-94, 44
Female sex – *n* (%)	34 (30)
Race – *n* (%)	
Chinese	82 (73)
Indian	11 (10)
Malay	7 (6)
Other	13 (11)
Family history of genetic disorder – *n* (%)	
Yes	29 (26)
No	59 (52)
Unknown	25 (22)
Location – *n* (%)	
Outpatient	107 (95)
Inpatient	6 (5)
Source of referral – *n* (%)	
Cardiology service	73 (65)
Adult Congenital Heart Disease (ACHD) service	11 (10)
Pediatrics service	11 (10)
Cardiovascular genetics (CVG) service	9 (8)
Cardiothoracic surgery (CTS) service	5 (4)
Others	4 (3)
Reason for referral – *n* (%)	
Diagnostic testing	83 (73)
Predictive testing	13 (12)
Segregation analysis	2 (2)
Continuation of care from another service	11 (10)
Review of genetic test results done elsewhere	4 (3)

### Source of referral and reason for referral

The most common source of referral was from the adult cardiology service (65%), followed by the adult congenital heart disease (ACHD) and pediatrics service. All, except one of the referrals from the pediatrics service, were for continuation of care or review of genetic results done elsewhere.

All patients who were referred by the CVG service were referred for predictive testing of asymptomatic at-risk relatives or segregation analysis.

Within the scope of diagnostic testing (*n* = 83), the most common reason for referral was for known or suspected genetic cardiomyopathies (73%) followed by aortopathies (12%). In the context of predictive testing (*n* = 13), the predominant indication was testing for hereditary transthyretin amyloidosis (hATTR) (*n* = 5) followed by cardiomyopathies (*n* = 4). The reason for referral based on known clinical or suspected clinical conditions is shown in Table 3.

**Table 3:** Reason for referral based on clinical/suspected clinical condition

Diagnostic testing (*n*)	83
Cardiomyopathy – *n* (%)	60 (73)
Hypertrophic cardiomyopathy or phenocopy	24
Dilated cardiomyopathy	18
ATTR cardiomyopathy	11
Hypokinetic non-dilated cardiomyopathy	4
Arrhythmogenic cardiomyopathy	3
Aortopathy – *n* (%)	10 (12)
Connective tissue disease – *n* (%)	6 (7)
Arrhythmogenic Disorder – *n* (%)	5 (6)
Long QT syndrome	3
Conduction disorder	1
Polymorphic ventricular tachycardia	1
Complex congenital heart disease – *n* (%)	1 (1)
Pulmonary arterial hypertension – *n* (%)	1 (1)
Predictive testing (*n*)	13
Cardiomyopathy – *n* (%)	12 (92)
ATTR cardiomyopathy	5
Dilated cardiomyopathy	4
Hypertrophic cardiomyopathy	2
Fabry disease	1
Aortopathy – *n* (%)	1 (8)
Marfan syndrome	1
Familial segregation analysis (*n*)	2
Aortopathy	2
Continuation of care (*n*)	11
Connective tissue disease	5
Aortopathy	3
Syndromic disorder	2
Cardiomyopathy	1
Review of genetic results elsewhere (*n*)	4
Chromosomal anomaly	3
ATTR cardiomyopathy	1

ATTR, transthyretin amyloidosis.

### Yield of genetic counseling and testing

Genetic testing was indicated and offered to 87% of the patients (98/113) referred to the CVG service. These were patients referred for diagnostic testing, predictive testing, or familial segregation analysis. After the pre-test and genetic counseling, 64% of the patients (63/98) proceeded with genetic testing (diagnostic testing *n* = 51, predictive testing *n* = 10, familial segregation analysis *n* = 2). The remaining 35 patients were undecided or declined genetic testing. The most cited reasons for declining genetic testing include concerns about cost, potential insurance implications, and a perceived lack of immediate benefit from testing. Out of 113 patients, 15 were not offered genetic testing either because it was done elsewhere (*n* = 11), or it was not indicated (*n* = 4) after review by the CVG team.

Of all patients who underwent genetic testing (*n* = 63), genetic variants were identified in 43 patients (68%), no variants were identified in 19 patients (30%) and 1 patient opted out of testing after the sample was sent to the laboratory. A pathogenic or likely pathogenic (P/LP) variant was identified in 22 patients (35%) and 20 patients (32%) had variant(s) of uncertain significance (VUS). Further review was undertaken for VUS reclassification.

After the reclassification of 3 VUS, 24 out of 63 (38%) patients had P/LP variants, and 15 out of 63 (24%) patients had VUS. Including all referrals to the CVG service, there were a total of 30 P/LP variants and 57 VUS. Table 4 details the American College of Medical Genetics and Genomics/Association for Molecular Pathology (ACMG/AMP) classification of variants seen in our cohort.

**Table 4:** ACMG/AMP classification of variants in our CVG service

Condition/Phenotype	Gene	Variant (GRCh38)	MANE Transcript ID	Variant (cDNA)	Variant (Protein)/ Predicted Effect	ACMG/AMP Criteria	ACMG/AMP Classification
HCM	ALPK3	NC_000015.10:g.84857665del	NM_020778.4	c.3533del	p.(Ser1178Metfs∗22)	PVS1 + PM2 + PP4	Pathogenic
	ALPK3	NC_000015.10:g.84857203A>G	NM_020778.4	c.3071A>G	p.(Gln1024Arg)	PM2 + BP4	VUS
	ALPK3	NC_000015.10:g.84839036T>C	NM_020778.4	c.967T>C	p.(Cys323Arg)	PM2	VUS
	DMD	NC_000023.11:g.32565836G>T	NM_004006.2	c.1858C>A	p.(Leu620Met)	PM2 + BP4	VUS
	DMD	NC_000023.11:g.32484961C>A	NM_004006.2	c.2761G>T	p.(Val921Phe)	PM2	VUS
	FHL1	NC_000023.11:g.136208576dup	NM_001159699.2	c.671dup	p.(Tyr224∗)	PVS1 + PM2 + PP4	Pathogenic
	FLNC	NC_000007.14:g.128840121G>A	NM_001458.4	c.1510 G>A	p.(Ala504Thr)	PM2	VUS
	FLNC	NC_000007.14:g.128835445A>C	NM_001458.4	c.472A>C	p.(Lys158Gln)	PM2 + PP3	VUS
	GAA	NC_000017.11:g.80108305C>T	NM_000152.3	c.971C>T	p.(Pro324Leu)	PM2 + PP3	VUS
	GAA	NC_000017.11:g.80104641G>T	NM_000152.3	c.55G>T	p.(Val19Leu)	PM2 + BP4	VUS
	RAF1	NC_000003.12:g.12584627A>T	NM_002880.3	c.1834T>A	p.(Ser612Thr)	PM2 + BP4	VUS
	RIT1	NC_000001.11:g.155900470A>C	NM_006912.5	c.578T>G	p.(Met193Arg)	PM2 + BP4	VUS
	RYR2	NC_000001.11:g.237660079G>A	NM_001035.2	c.8298+5G>A	-	PM2	VUS
	RYR2	NC_000001.11:g.237649902C>T	NM_001035.2	c.7538C>T	p.(Ala2513Val)	PM2 + PP3 + PM1	VUS
	TMEM43	NC_000003.11:g.(?_14166440)_(14185180_?)dup	NM_024334.3	-	-	PM2	VUS
	TNNT2	NC_000001.11:g.201359220C>T	NM_001276345.2	c.887G>A	p.(Arg296His)	PS4+PS3	Likely pathogenic (Reclassified from VUS)
	VCL	NC_000010.11:g.74090041A>C	NM_014000.3	c.1195A>C	p.(Asn399His)	BP4	VUS
Danon disease	LAMP2	NC_000023.11:g.120445855_120446402del	NM_002294.3	c.767_864+450del	p.(Asn257Tyrfs∗35)	PM2 + PVS1 + PP4	Pathogenic
Fabry disease	GLA	NC_000023.11:g.101398470A>G	NM_000169.2	c.899T>C	p.(Leu300Pro)	PM2 + PVS1 + PP4	Pathogenic
DCM/HNDC	ALPK3	NC_000015.10:g.84868248C>G	NM_020778.4	c.5516C>G	p.(Ala1839Gly)	BS1	VUS
	ALPK3	NC_000015.10:g.84859843C>T	NM_020778.4	c.4639C>T	p.(Arg1547Trp)	PM2	VUS
	ALPK3	NC_000015.10:g.84857634CTG[2]	NM_020778.4	c.3508_3510del	p.(Leu1170del)	PM2 + PM4	VUS
	DMD	NC_000023.11:g.(32448639_32454661)_(32645153_32697869)del	NM_004006.2	-	-	PM2 + PVS1 + PP4	Pathogenic
	DSP	NC_000006.12:g.7579768_7579769ins?	NM_004415.3	c.3578_3579ins?	p.(Lys1194fs)	PM2 + PVS1 + PP4	Pathogenic
	DSP	NC_000006.12:g.7555790del	NM_004415.3	c.243del	p.(Leu82∗)	PM2 + PVS1	Likely Pathogenic
	DSP	NC_000006.12:g.7581265_7581270del	NM_004415.3	c.5075_5080del	p.(Lys1692_Ser1693del)	PM2 + PM4	VUS
	EMD	NC_000023.11:g.154379737C>T	NM_000117.2	c.130C>T	p.(Gln44∗)	PM2 + PVS1 + PP4	Pathogenic
	GAA	NC_000017.11:g.80108766C>T	NM_000152.3	c.1264C>T	p.(Arg422Trp)	PM2 + BP4	VUS
	GATA5	NC_000020.11:g.62466538C>T	NM_080473.4	c.713G>A	p.(Arg238His)	PM2	VUS
	GALT	NC_000009.12:g.34646578GTCA[2]	NM_004415.3	c.-119_-116del	-	PM3 + PM4 + PS3 + PP4 + PP5	Pathogenic
	JUP	NC_000017.11:g.41769590G>A	NM_002230.2	c.296C>T	p.(Ser99Leu)	PM2 + BP4	VUS
	JUP	NC_000017.11:g.41767396C>T	NM_002230.2	c.892G>A	p.(Gly298Ser)	PM2 + PP3	VUS
	LZTR1	NC_000022.11:g.20990444G>A	NM_006767.3	c.710G>A	p.(Arg237Gln)	PM2 + BP4	VUS
	MYBPC3	NC_000011.10:g.47351427C>T	NM_000256.3	c.104G>A	p.(Arg35Gln)	BS1 + PP3	VUS
	MYH7	NC_000014.9:g.23415801C>T	NM_000257.3	c.4985G>A	p.(Arg1662His)	PM2 + BP4	VUS
	NF1	NC_000017.11:g.31206366G>A	NM _001042492.3	c.1387G>A	p.(Ala463Thr)	PM2 + BP4	VUS
	NF1	NC_000017.11:g.31259081T>C	NM_001042492.3	c.4382T>C	p.(Ile1461Thr)	PM2 + PP3	VUS
	PCCA	NC_000013.11:g.100330561G>A	NM_000282.3	c.1430G>A	p.(Gly477Asp)	BS1	VUS
	PCCB	NC_000003.12:g.136327706G>A	NM_000532.4	c.1372G>A	p.(Ala458Thr)	BS1 + PP3	VUS
	PCCB	NC_000003.12:g.136255922A>G	NM_000532.4	c.250A>G	p.(Met84Val)	BS1	VUS
	PKP2	NC_000012.12:g.32877984C>T	NM_001005242.3	c.896G>A	p.(Arg299His)	PM2 + BP4	VUS
	POU4F3	NC_000005.10:g.146339632G>A	NM_002700.3	c.205G>A	p.(Gly69Ser)	PM2	VUS
	RYR2	NC_000001.11:g.237590820C>T	NM_001035.2	c.3988C>T	p.(Pro1330Ser)	PM2	VUS
	SCN5A	NC_000003.12:g.38575424G>A	NM_000335.5	c.3536C>T	p.(Ala1179Val)	BS1 + BP4 + PP1 + PS3	VUS
	SLC22A5	NC_000005.10:g.132392538T>C	NM_003060.3	c.1373T>C	p.(Val458Ala)	PM2 + PP3	VUS
	SNX27	NC_000001.11:g.151639040A>G	NM_001330723.2	c.464A>G	p.(Tyr155Cys)	PM2	VUS
	SOS2	NC_000014.9:g.50150137A>T	NM_006939.2	c.2255T>A	p.(Phe752Tyr)	PM2 + BP4	VUS
	TTN	NC_000002.12:g.178612442G>A	NM_001267550.2	c.50083C>T	p.(Arg16695∗)	PM2 + PVS1 + PP5	Pathogenic
	TTN	NC_000002.12:g.178589316C>T	NM_001267550.2	c.62409G>A	p.(Trp20803∗)	PM2 + PVS1	Likely Pathogenic
	TTN	NC_000002.12:g.178569854C>T	NM_001267550.2	c.76278G>A	p.(Trp25426∗)	PM2 + PVS1	Likely Pathogenic
ATTR-CM	TTR	NC_000018.10:g.31595221C>T	NM_00371.4	c.302C>T	p.(Ala101Val)	PS4 + PP4 + PP5	Likely Pathogenic (Reclassified from VUS)
	TTR	NC_000018.10:g.31598580G>T	NM_000371.4	c.349G>T	p.(Ala117Ser)	PS4 + PS3 + PP3	Pathogenic
	TTR	NC_000018.10:g.31598655G>A	NM_000371.4	c.424G>A	p.(Val142Ile)	PS4 + PS3 + PP3	Pathogenic
ACM/ARVC	PKP2	NC_000012.12:g.32878336del	NM_001005242.3	c.544del	p.(Glu182Lysfs∗8)	PM2 + PVS1 + PP4	Pathogenic
Arrhythmogenic disorders	CDH2	NC_000018.10:g.27985576A>C	NM_001792.4	c.1927T>G	p.(Leu643Val)	PM2 + BP4	VUS
Aortopathy	COL5A1	NC_000009.12:g.134802993A>G	NM_000093.4	c.3112A>G	p.(Lys1038Glu)	BS1	VUS
	FBN1	NC_000015.10:g.48505019T>A	NM_000138.4	c.1960+6A>T	-	PM2 + BP4	VUS
	FBN1	NC_000015.10:g.48468483T>C	NM_000138.4	c.4511A>G	p.(Asn1504Ser)	PM2 + PP2 + PP3 +PP4 + PP5	Likely Pathogenic
	FBN2	NC_000005.10:g.128345413C>A	NM_001999.3	c.3161G>T	p.(Arg1054Leu)	BS4 + BP4	Likely benign (Reclassified from VUS)
	MYLK	NC_000003.12:g.123737484C>A	NM_053025.3	c.648G>T	p.(Gln216His)	PM2 + BP4	VUS
	PLOD1	NC_000001.11:g.11964188C>T	NM_000302.3	c.1216C>T	p.(Pro406Ser)	BS1 + PP3	VUS

Includes P/LP/VUS and reclassified variants; excludes cases where genetic testing was done elsewhere.

ACM, arrhythmogenic cardiomyopathy; ACMG, American College of Medical Genetics and Genomics; AMP, Association for Molecular Pathology; ARVC, arrhythmogenic right ventricular cardiomyopathy; ATTR-CM, transthyretin amyloid cardiomyopathy; cDNA, complementary DNA; CVG, cardiovascular genetics; DCM, dilated cardiomyopathy; HCM, hypertrophic cardiomyopathy; HNDC, non-dilated cardiomyopathy; LP, likely pathogenic; MANE, Matched Annotation; P, pathogenic; VUS, variant of uncertain significance.

Out of the total 73 referrals to the CVG service, encompassing cases where genetic testing was both indicated and conducted, the genetic testing results, regardless of their specific outcomes (P/LP, VUS or LB/B [likely benign/benign]), added value to the care of 44 patients (60%) in one or more these ways: clarifying an established or suspected clinical diagnosis, ruling out the presence of inherited cardiac condition (for predictive testing), aiding in VUS resolution, resulting in changes clinical management (eg, guiding clinical surveillance strategy, guiding threshold for implantable cardioverter-defibrillator insertion for primary prevention of sudden cardiac death in genetic cardiomyopathy, the timing of prophylactic surgery intervention in aortopathy, etc.) and/or cascade testing.

### Diagnostic genetic testing results by clinical/suspected clinical condition

The most frequent reason for referral to diagnostic genetic testing was suspicion of established cardiomyopathy (Table 3). Within this category, a significant focus (*n* = 24) was on distinguishing between hypertrophic cardiomyopathy (HCM) and phenocopies. Four patients were found to have VUS, 1 patient had no variants identified, and 1 patient opted out of testing after the sample was sent to the laboratory. Among the remaining 5 patients, 1 individual received a diagnosis of Fabry disease, another patient was diagnosed with Danon disease, another patient was diagnosed with a novel *FHL1* (HGNC:3702, NM_001159699.2)-related X-linked HCM with X inactivation, 1 patient was diagnosed with a novel *ALPK3* (HGNC:17574, NM_020778.4)-related HCM, whereas another was diagnosed with *TNNT2* (HGNC:11949, NM_001276345.2)-related HCM, which is notably more prevalent in the local population.

Genetic testing was conducted in 14 out of 18 patients referred for dilated cardiomyopathy. P/LP variants were identified in 6 patients – 3 patients were diagnosed with *TTN* (HGNC:12403, NM_001267550.2)-related cardiomyopathy, 1 patient was diagnosed with *DSP* (HGNC:3052, NM_004415.3)-related cardiomyopathy, 1 patient was diagnosed with X-linked Emery-Dreifuss cardiomyopathy with mild muscular dystrophy, and 1 patient was diagnosed with *DMD* (HGNC:2928, NM_004006.2)-related Becker Muscular Dystrophy.

Two out of 4 patients referred for hypokinetic non-dilated cardiomyopathy (HNDC) underwent genetic testing. Among these patients, 1 individual presented with multiple congenital defects and partial agenesis of the corpus callosum in addition to HNDC. The decision was made for broader testing with exome sequencing and mitochondrial genome testing. The results indicated a likely pathogenic (LP) variant in the *DSP* gene supporting a diagnosis of arrhythmogenic left ventricular cardiomyopathy as the cause of the patient’s HNDC. However, no genetic basis was identified for the patient's congenital malformations. The other patient had a VUS.

All 11 patients referred for hATTR diagnostic testing had an established diagnosis of transthyretin amyloid cardiomyopathy (ATTR-CM) based on cardiac imaging features suggestive of ATTR-CM, absence of monoclonal protein and a positive ^99m^technetium-pyrophosphate bone scintigraphy scan. Nine out of 11 patients proceed with single gene (*TTR*) testing after counseling. P/LP variants were identified in 4 patients thereby establishing a diagnosis of hATTR and facilitating cascade testing for at-risk family members. No variants were seen in the remaining 5 patients – 4 patients were diagnosed with wild-type ATTR amyloidosis, and 1 patient was referred to the neurology service for evaluation of possible mitochondrial disorder in view of constellation of other findings such a peripheral neuropathy, retinitis pigmentosa, and hearing impairment.

All 3 patients referred for diagnostic testing for arrhythmogenic cardiomyopathy fulfilled the modified task force criteria for arrhythmogenic right ventricular cardiomyopathy. One patient was diagnosed with *PKP2* (HGNC:9024, NM_001005242.3)-related arrhythmogenic right ventricular cardiomyopathy, VUS was identified in 1 patient and the other patient declined genetic testing.

Three out of 5 patients referred for arrhythmogenic disorders underwent genetic testing. One patient with suspected long QT syndrome was found to have a VUS, whereas no variants were found in the other patient. Genetic testing yielded no variants for the patient exhibiting conduction disorder with intermittent high-grade atrioventricular block.

Six out of 10 patients referred for aortopathy opted for genetic testing. P/LP variants in *FBN1* (HGNC:3603, NM_000138.4) were identified in the 2 patients who also fulfilled the revised Ghent criteria for Marfan syndrome. One patient had VUS, another patient had an LB/B variant ascertained through familial segregation testing and no variants were seen in the remaining 2 patients. These 4 patients did not fulfill the clinical criteria for Marfan syndrome.

Six patients were referred for suspected connective tissue disease (CTD) without aortopathy. Clinical findings such as skin laxity, joint hypermobility, spontaneous pneumothorax, tall stature, or polyvalvular regurgitation prompted these referrals. After review, genetic testing was not indicated in 3 cases, and in the remaining 3 cases, no variants were found on genetic testing.

The only patient who was referred for complex congenital heart disease was offered genetic testing; however, he was undecided.

For the patient who was referred for assessment of hereditary pulmonary arterial hypertension, no variant was identified.

### Predictive genetic testing results by clinical condition

Five asymptomatic family members of probands with hATTR underwent predictive genetic testing. A P/LP variant in the *TTR* (HGNC:12405, NM_00371.4) gene was identified in 3 patients and no variants were reported in the remaining 2 patients, releasing the latter from lifelong ATTR-CM surveillance.

Predictive testing for dilated cardiomyopathy was done in 4 asymptomatic patients. One patient was found to be heterozygous for a pathogenic variant in *TNNT2* and, another patient was found to have an X-linked *DMD* variant that is associated with Becker Muscular Dystrophy. Family-specific variants were not seen in the other 2 patients.

None of the cases referred for predictive testing for HCM (*n* = 2) and Fabry disease (*n* = 1) had genetic testing done. One of the referrals was deemed to be inappropriate (the patient’s father is the HCM proband) and the other patients were undecided.

A positive result was obtained from predictive testing for a likely pathogenic *FBN1* variant in a patient whose son was diagnosed with Marfan syndrome-related aortic dissection. The patient herself did not have overt syndromic features or ectopia lentis and had a low systemic score. She was referred for further cardiac evaluation.

### VUS reclassification

After clinical and literature review, 3 VUS were reclassified. In 1 case of diagnostic testing for hATTR, a VUS in *TTR* c.302C>T p.(Ala101Val) was reclassified as LP by the clinical laboratory after review. This missense variant is not reported in population databases (gnomAD), but it has been reported in other patients with hATTR, and advanced modeling of the protein sequence is expected to disrupt *TTR* protein function.^18^^,^^19^ Therefore the classification of the variant using ACMG criteria was revised to LP (PS4 + PP4 + PP5).

In another case of diagnostic testing for apical HCM, *TNNT2* c.857G>A (p.Arg286His) variant was reported as VUS. However, it was reclassified as LP. Based on ACMG criteria (PS4 + PS3). This reclassification was supported by evidence showing that the variant is enriched in the Singaporean HCM cohort, albeit with low penetrance, and by induced pluripotent stem cells functional study demonstrating cellular manifestations of HCM associated with this variant.^20^

In a patient with a dilated aortic root, the aortopathy panel revealed a VUS in *FBN2* (HGNC:3604, NM_001999.3) c.3161G>T (p.Arg1054Leu). The patient however did not have any clinical features of Beal syndrome, a *FBN2*-related disorder. Familial segregation analysis in 2 phenotypically unaffected first-degree family members revealed they too had the same VUS. This was suggestive that the variant was less likely to be disease-causing. This VUS was subsequently reclassified from VUS to LB status by the clinical laboratory (BS4 + BP4).

## Discussion

### Multidisciplinary collaboration in CVG service

Integration of CVG genetics into clinical practice is a pivotal advancement in the field of cardiology, offering a refined approach to patient care. Our pilot initiative underscores the feasibility and clinical benefit of incorporating CVG care as standard practice within NHCS, a tertiary cardiovascular center, in collaboration with SDGMC.

Before the implementation of our pilot service, CVG practice was fragmented with specialists working within their own areas of interest. Our service has improved the fragmented landscape by leveraging the expertise of cardiologists with an interest in genetics, geneticists with an interest in cardiology, genetic counselors, and researchers. This collaborative setup has encouraged the exchange of knowledge and facilitated the development of procedural frameworks that are crucial for clinical practice. For instance, patients who were diagnosed with genetic cardiomyopathies with multiorgan involvement would be subsumed under the care of a specialist team that would offer comprehensive care, a stark contrast to the uncoordinated care they had previously received. Another example is where standardized disease and variant-specific memos were created for probands to share with their family members to facilitate cascade testing and increase accessibility to the clinical CVG service. Future work will aim to test the feasibility of protocolizing these pilot frameworks and downstream evaluation of these protocols including their impact on clinical decision-making in CVG and patient outcomes is warranted.

Over a span of 2.5 years, the volume of referrals to our CVG service doubled (Figure 1). This upward trend reflects the growing recognition of the benefits of genetic testing within the cardiology community and its increasing acceptance among patients.^21^ The increase in referral volume also coincided with the addition of a cardiologist to the CVG service emphasizing the need for collaboration with domain-specific specialists to drive a genetic service within any discipline of medicine.^22^

With the growing utilization of CVG testing, exercising clinical discretion regarding the appropriateness of genetic testing and the type of genetic testing is of paramount importance. This level of decision-making is best achieved through a collaborative approach, with a combined clinic that includes a cardiologist, geneticist, and genetic counselor. The benefit of this was demonstrated in cases where patients were not recommended genetic testing after review by the CVG team, as it was deemed inappropriate. In 1 case, the patient referred was the son of an individual with HCM. In this instance, genetic testing of the proband, ie, the father with a definite phenotype would yield more meaningful insights. For the other patients who were not well “phenotyped”, clinical surveillance rather than genetic testing was recommended. This approach was chosen owing to a lower yield of genetic testing and greater uncertainty of the latter with an unclear phenotype.^23^ Practice guidelines place a strong emphasis on rigorous disease-appropriate phenotyping and 3-generation pedigree analysis to increase the yield of identifying a disease-causing variant.^3^^,^^5^

With the establishment of our clinical CVG service, we have forged stronger connections with the basic and translation research teams at our center. The synergy between the clinical and research teams holds great potential as it enables research endeavors to effectively address the unmet clinical needs observed in our patient population.^24^ One such example is the work we have undertaken in variant resolution in HCM patients using a multi-modal platform that combines cutting-edge artificial intelligence models with human cellular models.

### Clinical impact of our CVG service

In a substantial number of patients who underwent genetic testing (60%), it offered clarity regarding an existing or suspected clinical diagnosis or, eliminated the possibility of inherited cardiac conditions, or aided in VUS resolution. Although cascade testing among at-risk family members traditionally is thought to be the motivation for proband genetic testing,^25^^,^^26^ the emergence of targeted therapies for cardiovascular conditions (eg, ATTR-CM, Fabry disease) has amplified the importance of establishing a genetic diagnosis. Furthermore, this approach has enriched our comprehension of the interplay between phenotypes and genotypes in cardiovascular disease. Consequently, we are now more adept at explaining disease trajectory, prognosis, and management options to affected individuals with greater precision.^27^

In our cohort, this was best appreciated in patients referred for diagnostic testing for cardiomyopathies. For instance, we identified 2 patients below the age of 30, with rare HCM phenocopies: Danon disease and Fabry disease due to pathogenic variants in the *LAMP2* (HGNC:6501, NM_002294.3) gene and *GLA* (HGNC:4296, NM_000169.2) gene, respectively. Notably, these conditions follow distinct disease courses and necessitate different treatment approaches compared to HCM. Similarly, within the cohort of cardiomyopathy patients, the identification of arrhythmogenic variants within *DSP*, *PKP2,* and *EMD* (HGNC:3331, NM_000117.2) genes prompted discussions or further validated the decision regarding implantable cardioverter-defibrillator insertion for primary prevention of sudden cardiac death. This stems from the fact that these genotypes are associated with a significantly higher risk of malignant arrhythmias.^3^^,^^28^ In patients with established clinical and imaging diagnoses of ATTR-CM, genotyping was essential to differentiate between hATTR and wild-type transthyretin amyloidosis to facilitate cascade testing in 5 at-risk family members.^29^ In this context, adopting a family-specific variant-guided approach was pivotal to ascertaining the genetic predisposition of at-risk family members. This, in turn, expedited cardiac imaging, and where positive, initiation of early treatment with targeted therapy was discussed. TTR protein stabilizer, Tafamidis has been shown to have greater survival benefit when administered at earlier stages of cardiac disease.^30^ Without the inclusion of genetic testing, attaining conclusive diagnoses for these cases would have remained challenging, potentially leading to suboptimal management.

Although further research is needed to establish the full extent of the impact of CVG on cardiovascular outcomes, existing studies suggest that it proves to be cost-effective in the context of cascade testing, by negating the need for lifelong clinical and imaging surveillance in genotype-negative family members.^31^, ^32^, ^33^ Apart from the cost-saving aspect, it is important to emphasize the sense of relief observed in our cohort of patients who received favorable predictive testing results. On the other hand, asymptomatic family members who were found to carry P/LP variants in genes like *TTR*, *DMD*, *TTN*, and *FBN1* received psychosocial support, were connected with support groups, and were referred for surveillance to guide their clinical management. These issues were comprehensively addressed in the post-test genetic counseling sessions. Acknowledging the emotional burden of receiving a diagnosis when asymptomatic, especially considering the uncertainty surrounding disease manifestation due to incomplete and/or age-related penetrance and variable expressivity, our CVG team ensured ongoing accessibility via email or phone to address any concerns or inquiries that might arise outside of scheduled clinic visits.

### Potential and prospects in CVG

Our CVG service is intricately linked to the Singapore National Precision Medicine program, which is leading the transformation in healthcare provision through the development and analysis of large-scale genomic-phenotypic databases, with a specific focus on the Asian population, which remains underrepresented in genomic databases.^34^^,^^35^ In fact, a substantial proportion of our patients who underwent genetic testing yielded VUS, which is not clinically actionable and can potentially contribute to increased psychological distress.^36^ A local study showed that Singaporeans with HCM had 3 times as many VUS in HCM-related genes compared to patients of European descent.^20^ These observations underscore the scarcity of racial and ethnic diversity in genomic repositories, leading to a higher prevalence of inconclusive results among individuals of Asian ancestry.^37^ By advancing our CVG initiative, we hope to actively contribute to the growth of an inclusive and regionally relevant genomic database, which, in turn, will facilitate a deeper comprehension of the distinctive genetic risks linked to underrepresented Singaporean and Asian racial and ethnic diversities. Ultimately, our overarching objective is to collaborate closely with governmental and regulatory bodies to transform these insights into practical clinical applications and shape healthcare policies resulting in both cost-effective care and meaningful health care outcomes.

Our pilot initiative has also highlighted the existing gaps in education and training needed to keep pace with advancements in cardiovascular genomics. Recognizing the significance of genomics in modern medicine, scientific bodies have advocated for a dedicated CVG training program.^22^^,^^24^^,^^38^ Clinical CVG, as a subset of precision medicine, is a nascent field with burgeoning interest within Singapore, notably with the establishment of the Singapore National Precision Medicine program. However, there is currently a lack of a structured training program to equip clinicians with the required core competency in this field. This presents a valuable opportunity to curate a clinical CVG program or rotation that can be seamlessly integrated into existing residency programs. Through this initiative, residents not only gain early exposure to clinical CVG but also develop a deeper understanding of the complexities surrounding CVG, including its ethical, social, and legal implications. The early exposure to the benefits and constraints of clinical CVG holds great potential to inspire residents to diversify their interests beyond clinical practice. This encompasses various domains such as genomic research, basic science, advanced statistics, and health care policy, all of which can lead to valuable contributions that ultimately improve patient care.^39^

## Conclusion

By embarking on our pilot CVG service, we have developed a heightened understanding of the implications and constraints associated with genetic testing within cardiovascular care. Additional effort is needed to reduce barriers to clinical CVG testing within our cohort, specifically addressing cost concerns, mitigating potential insurance discrimination, and streamlining the referral infrastructure for cascade testing.

We have also acquired insights into the essential infrastructure and operational requirements needed to maintain a sustainable and clinically effective service. Moreover, our collaboration with various stakeholders has deepened and is poised for further strengthening. This has elevated the profile of clinical CVG among healthcare professionals and patients and, has provided us with the foundation to actively participate in national precision medicine initiatives, which aim to treat and prevent cardiovascular diseases. We are committed to further enhancing and expanding our CVG service and will strive to realize the full potential of cardiovascular genomics to improve healthcare outcomes.

## Data Availability

The data used in this study are not publicly available due to privacy and legal restrictions but are available from the corresponding author on request.

## Conflict of Interest

The authors declare no conflicts of interest.
